# Identification of occult metastases of medullary thyroid carcinoma by pentagastrin-stimulated intravenous calcitonin sampling followed by targeted surgery

**DOI:** 10.1111/j.1365-2265.2007.02747.x

**Published:** 2007-03-01

**Authors:** Matthias Schott, Holger S Willenberg, Cornelia Sagert, Thi-Bang-Tam Nguyen, Sven Schinner, Mathias Cohnen, Kenko Cupisti, Claus F Eisenberger, Wolfram T Knoefel, Werner A Scherbaum

**Affiliations:** * Department of Endocrinology, Diabetes and Rheumatology Germany; † Institute of Diagnostic Radiology Germany; ‡ Department of General and Visceral Surgery, University Hospital Duesseldorf Germany

## Abstract

**Background:**

High calcitonin (CT) serum levels suggest metastatic spread in medullary thyroid carcinoma (MTC) after thyroidectomy. In limited disease stages, however, morphological investigations including ultrasound, magnetic resonance imaging (MRI) and 18F-FDG positron emission tomography ([18F]FDG-PET) may often fail to identify exact tumour sites.

**Objective:**

The aim of the present study was to establish an improved strategy to identify small cervical tumours by combining pentagastrin stimulation with bilateral cervical intravenous CT sampling followed by high-resolution ultrasound.

**Design and patients:**

Six MTC patients were examined, of whom five patients already had bilateral neck dissection. Five patients had sporadic MTC, and one patient suffered from MEN2a.

**Results:**

Retrospective analysis of all patients revealed a highly sensitive positive correlation between an early calcitonin peak (20–40 s after pentagastrin injection) and site of cervical tumour affection. Postinterventional ultrasound examination of the affected regions of the neck revealed suspicious presence; in some cases small lymph nodes of less than 1 cm in size were then surgically excised. On histology, small tumours could be identified in four patients. Postsurgical examination revealed a clear decline of basal serum calcitonin levels in four patients (between −41% and −100%). In two patients CT normalized to baseline levels (< 10 pg/ml) and in another two patients CT rendered to near normal (14 and 17 pg/ml).

**Conclusion:**

Pentagastrin stimulation-based intravenous catheter sampling may be beneficial in the diagnostic work-up of MTC after thyroidectomy. Our data show that an early calcitonin peak (20–40 s after administration of pentagastrin) helps to identify tumour-affected regions.

## Introduction

Medullary thyroid carcinoma (MTC) is a rare tumour derived from the parafollicular calcitonin (CT)-secreting cells of the thyroid, explaining the key role of CT as a specific and sensitive marker of this cancer. MTC occurs in the sporadic form in about 70–80% of cases, while the remaining 20–30% are due to inherited forms^1^ including either isolated (familial MTC) or part of multiple endocrine neoplasia syndromes. Prognosis of MTC is relatively good with a 10-year survival rate ranging from 47% to 78%^2^,^3^ and even better in patients with CT doubling-time of more than 2 years.^4^ Within the past decade the prognosis has improved mainly because of earlier diagnosis and improvement in surgical procedures.^5^,^6^ Nevertheless, more than 50% of nonprophylactic thyroidectomized patients are not cured after surgery.^5^ In these cases residual tumour cells can be detected by measuring serum calcitonin alone or in combination with pentagastrin stimulation. In limited disease stages imaging studies, including ultrasound and MRI often fail to identify affected organs. [18F]FDG-PET has recently been proposed to identify residual tumour masses.^7^,^8^ Nonetheless, this procedure also has limitations as small tumours are rarely detected. Conventional intravenous calcitonin sampling may identify tumour-affected regions with larger tumour burden resulting in moderately to highly elevated serum calcitonin levels; however, no micrometastases with slightly increased calcitonin levels can be identified either.^6^,^9^–^12^

The aim of the present study was to investigate the prognostic value of a combined diagnostic strategy to identify tumour-affected areas. We here performed intravenous catheter CT sampling of potentially affected areas with pentagastrin stimulation in parallel followed by high-resolution ultrasound. On the basis of this approach we could identify tumour-affected cervical regions that could then be surgically revised.

## Subjects and methods

### Subjects

Clinical data of all patients are summarized in Table 1. Five patients with occult metastatic spread of previously diagnosed MTC were examined. In these patients persistent MTCs were diagnosed by elevated basal serum CT levels (normal < 10 pg/ml without previous thyroidectomy) and pathological stimulation after administration of pentagastrin (except Patient 5 with highly elevated basal CT levels). In all of these patients distant metastases were excluded by performing computed tomography. Patient 4 suffered from multinodular goitre with slightly elevated basal calcitonin levels including pathological stimulation of CT after administration of pentagastrin. All but one patient suffered from sporadic MTC. Patient 6 had multiple endocrine neoplasia type 2a. This patient had suffered from bilateral pheochromocytoma which had been surgically removed before. Five patients (1, 2, 3, 5 and 6) already had, in some cases multiple, bilateral neck dissections and lymphadenectomies. All previous operations were performed by experienced surgeons, most of them at the Department of Surgery at the University Hospital Duesseldorf in Germany.

**Table 1:** Patient characteristics

					Before operation		After operation	
Patient	Age (years), gender	Initial diagnosis	Type of MTC	Previous treatments	Basal CT	Stimulated CT	Basal CT	Stimulated CT
1	59, female	1998	sporadic	TE, ND, LE(1)	48 pg/ml	431 pg/ml	47 pg/ml	579 pg/ml
2	41, female	1989	sporadic	TE, ND, LE(2)	23 pg/ml	272 pg/ml	14 pg/ml	134 pg/ml
3	38, female	2003	sporadic	TE, ND, LE(3)	41 pg/ml	527 pg/ml	17 pg/ml	242 pg/ml
4	64, male	2004	sporadic	none	17 pg/ml	155 pg/ml	< 5 pg/ml	< 5 pg/ml
5	39, female	1994	sporadic	TE, ND, LE(5)	226 pg/ml	n.d.	7 pg/ml	26 pg/ml
6	44, male	1998	MEN2a	TE, ND	10 pg/ml	700 pg/ml	31 pg/ml	1347 pg/ml

Type of medullary thyroid carcinoma (MTC): MEN2a, multiple endocrine neoplasia type 2a.

Previous treatments: TE, thyroidectomy; ND, bilateral neck dissection; LE, additional cervical lymphadenectomy (number of additional LEs); CT, calcitonin.

### Methods

#### Pentagastrin-stimulated calcitonin sampling

Pentagastrin-stimulated intravenous calcitonin sampling was performed before surgical intervention. In each patient two intravenous catheters were placed in the regions of interest, mostly in lower parts of both internal jugular veins. Because of altered anatomy in Patient 5, both catheters were placed in the brachiocephalic veins. Body weight-adapted (0·5 µg per kg body weight) pentagastrin stimulation (Wallsend, Cambridge, UK) was then performed using peripheral blood sampling for control at baseline and 20 s, 40 s and 60 s after peripheral pentagastrin stimulation and thereafter every 30 s for 4 min. Since an interim analysis of Patient 1, 2 and 3 showed that sufficient interpretation can be reached by studying time-points for CT measurement within the first 120 s, the procedure was shortened in Patient 4, 5 and 6. In parallel, peripheral blood CT assessment was performed in all patients for comparison.

#### Calcitonin assay

A solid-phase, enzyme-labelled, two-site chemiluminescent immunometric assay was used for measurement of calcitonin. The assay was performed as recommended by the manufacturer (Immulite 2000, DPC, Bad Nauheim, Germany). On the basis of this method only the intact mature form of CT was detected. The detection level of the assay is 2 pg/ml. The intra and interassay coefficients of variation were between 2·8% and 6·2%, and 3·3 and 6·9%, respectively, for values ranging from 11·5 up to 1628·0 pg/ml.

## Results

The pentagastrin stimulation-based intravenous CT sampling was performed in six patients. Initially, the sampling was performed over a time period of 270 s (Patient 1, 2 and 3). This procedure was shortened to 120 s in Patient 4, 5 and 6. In all patients an early CT peak could be detected on one cervical site at 20 s and at maximum 40 s after peripheral intravenous administration of pentagastrin. This cervical region was then considered as highly suspicious for presence of remnant tumour cells. High-resolution ultrasound of the potentially affected cervical region was then performed by an experienced clinician and suspicious nodules were marked. In most cases, these nodules were only few millimeters in size (< 10 mm). Because of the size and other anatomical considerations, fine needle aspiration was not an option to be performed. Subsequently potentially affected regions in all patients were surgically revised.

Based on the histological results, small MTCs were found in 4 out of 6 patients (Patients 2, 3, 4 and 5). All results correlated with the presurgical predicted site of potential cervical tumour affection. In these four patients a decline of basal serum CT could be detected (−39% up to −97%). Comparable results were seen in Patient 2, 3 and 4 after intravenous stimulation with pentagastrin (−34% up to −100%). In one patient (Patient 4) the tumour could be completely removed, whereas in Patient 5 only a slight increase of serum calcitonin could be detected after stimulation with pentagastrin (maximum serum calcitonin 26 pg/ml). This is crucial, for instance as in Patient 5, no complete tumour exstirpation could be achieved even after six operations. In Patient 4 with a multinodular goitre, without any previous cervical operations we could predict where the tumour was located in the thyroid lobe. In Patient 2 and 3, the positive histology correlated with a decline of postinterventional basal (−39% up to −59%) and stimulated calcitonin. However, CT levels were still detectable after surgery. In Patient 6 with borderline basal serum CT levels, neither tumour cells could be detected on histology and nor did serum CT levels decline after surgical intervention. The same observations were made in Patient 1 who had more elevated basal CT levels.

## Discussion

To the best of our knowledge, this is the first description of an in general simple method to localize occult metastatic spread of medullary thyroid carcinoma (MTC) by combining separated intravenous calcitonin sampling in parallel with pentagastrin stimulation. Our data clearly show that an early calcitonin peak (about 20–40 s after intravenous pentagastrin administration) collected in the lower part of both jugular veins is a very strong indicator of residual tumour tissue at the cervical site where the catheter is placed. Having these results in mind a high-resolution ultrasound was performed in each case by an experienced clinician and suspicious cervical nodules were marked. Then targeted surgery by an experienced surgeon was performed. Based on this approach, residual tumours could be identified in four of six patients leading to a pronounced decrease of serum calcitonin. To evaluate the efficacy of this procedure peripheral pentagastrin stimulation was also performed after surgical intervention. On that basis, two of our patients could be cured ‘biochemically’ as judged by basal serum calcitonin measurements. After pentagastrin stimulation one of the two patients showed no increase in serum calcitonin, whereas the other patient revealed a slight increase. These results are absolutely crucial as some of our patients had already undergone diverse surgical interventions as in Patient 5, who had already experienced six cervical operations including one bilateral neck dissection and five lymphadenectomies.

A multitude of different imaging methods have already been studied for the detection of residual tumour tissue in MTC. A fairly good sensitivity of 93% with a specificity of 75% was achieved by MRI for differentiation of a scar from recurrent tumour disease in the thyroid bed.^13^ Scintigraphical methods have also been studied including pentetreotide scintigraphy with a reported sensitivity between 37% and 71%, respectively.^14^–^17^ Comparable results were also seen using ^99m^Tc-V-DMSA with a sensitivity of 68% for imaging MTC metastases^18^ as well as for CEA-antibodies labelled with ^111^In or ^99m^Tc with sensitivities of around 60%.^19^,^20^[18]FDG PET may play a complementary role in tumour detection.^7^ The most sensitive method for localization of a recurrent tumour mass or of lymph node metastases in the neck is, however, conventional high-resolution ultrasound which gives a sensitivity of around 96% and a specificity of 83%.^21^ Nonetheless, if the maximum tumour size is a few millimeters only and there are only slightly to moderately increased serum CT levels, the tumours might not be detected by this method. Conventional selective venous catheterization may represent an additional method to guide the surgeon to the cervical site that has to be operated on. This method, however, only works if the tumour is large enough for detection of differences in serum CT on a basal level.^9^ In patients with small tumour residues this method will fail to detect differences. Using the combined strategy described here, there is a fairly good chance to determine the cervical site on which the surgeon should operate. Of note, one of our patients (Patient 4) had never undergone previous cervical operation and had just slightly increased serum CT levels along with a pathological CT stimulation at the time of presentation. Because of the patient's wish, the procedure was also offered to him after receiving informed consent. Based on our results, we were able to predict which thyroid lobe was affected by the tumour. If these data are confirmed in a larger patient population with sporadic MTC, operations strategies for very small tumours (with only slightly elevated CT levels) might be altered in the future. This procedure should, however, only be offered to patients with slightly elevated basal and stimulated CT levels, and not to patients with markedly increased CT levels or multinodular goitre. Without an exact answer to this question, total thyroidectomy along with bilateral lymph node dissection will remain the therapy of choice.

**Fig. 1:**
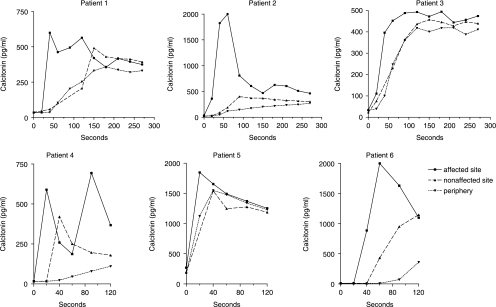
Serum calcitonin levels after intravenous administration of pentagastrin.

In summary, the method described here may represent an alternative approach to identify tumour-affected cervical regions in patients with metastasized MTC. This method may, however, only be successful if the tumour is confined to the neck on one cervical site and with slightly to moderately elevated serum calcitonin levels only. In patients with higher basal serum CT levels, differences might not be detected. Based on our results we can recommend our work-up strategy if patients have maximum calcitonin values of 100 pg/ml to 250 pg/ml. As mentioned above, the procedure itself is simple to plan and the technical equipment is not sophisticated, as it only combines intravenous catheter sampling with pentagastrin stimulation followed by high-resolution ultrasound. On the other hand, the blood drawings at three different localizations (the periphery and both jugular veins or brachiocephalic veins, respectively) need to be performed within precise time limits. To our experience, this can only be performed with three physicians performing blood sampling and another three persons assisting blood sampling. This might, however, be worthwhile in patients where no exact identification of tumour areas has been reached despite a combination of several imaging techniques (Fig. 1).

Calcitonin (CT) sampling was performed after pentagastrin stimulation at both jugular veins and the periphery for comparison. Because of altered anatomy, blood sampling in Patient 5 was performed from both brachiocephalic veins as well as the periphery. Initially, the sampling was performed over a time period of 270 s (Patient 1, 2 and 3). The procedure was shortened to 120 s in Patients 4, 5 and 6. In all patients an early CT peak could be detected on one cervical site at 20 s and at maximum 40 s after peripheral intravenous administration of pentagastrin. This cervical region was then considered as highly suspicious for presence of remnant tumour cells.
